# *Bifidobacterium animalis* subsp. *lactis* A6 Alleviates Obesity Associated with Promoting Mitochondrial Biogenesis and Function of Adipose Tissue in Mice

**DOI:** 10.3390/molecules25071490

**Published:** 2020-03-25

**Authors:** Yanxiong Huo, Xuhong Lu, Xiaoyu Wang, Xifan Wang, Lingli Chen, Huiyuan Guo, Ming Zhang, Yixuan Li

**Affiliations:** 1 Beijing Advanced Innovation Center for Food Nutrition and Human Health, College of Food Science & Nutritional Engineering, China Agricultural University, Beijing 100083, China; huoyanxiong195615@163.com (Y.H.); wangxfan@126.com (X.W.); chenlingli89@163.com (L.C.); 2 Key Laboratory of Functional Dairy, Co-constructed by ministry of Education and Beijing Municipality, College of Food Science & Nutritional Engineering, China Agricultural University, Beijing 100083, China; luxuhong@cau.edu.cn (X.L.); xy.wang@cau.edu.cn (X.W.); 3 Beijing Laboratory of Food Quality and Safety, College of Food Science and Nutritional Engineering, China Agricultural University, Beijing 100083, China; guohuiyuan@cau.edu.cn; 4 School of Food and Chemical Engineering, Beijing Technology and Business University, Beijing 100048, China

**Keywords:** *Bifidobacterium animalis* subsp. *lactis* A6, obesity, mitochondrial biogenesis and functions, lipopolysaccharides, tumor necrosis factor α

## Abstract

Probiotics are widely known for their health benefits. Mitochondrial dysfunction is related to obesity. The aim of this study was to illuminate whether *Bifidobacterium animalis* subsp. *lactis* A6 (BAA6) could improve obesity due to increased mitochondrial biogenesis and function of adipose tissues. Four-week-old male C57BL/6 mice were fed with a high-fat diet (HFD) for 17 weeks. For the final eight weeks, the HFD group was divided into three groups including HFD, HFD with BAA6 (HFD + BAA6 group), and HFD with *Akkermansia muciniphila* (AKK) (HFD + AKK group as positive control). The composition of the microbiota, serum lipopolysaccharides (LPS), and mitochondrial biosynthesis and function of epididymal adipose tissues were measured. Compared with the HFD group, body weight, relative fat weight, the relative abundance of *Oscillibacter* and *Bilophila*, and serum LPS were significantly decreased in the HFD + BAA6 and HFD + AKK groups (*p* < 0.05). Furthermore, the addition of BAA6 and AKK increased the expression of peroxisome proliferator-activated receptor γ coactivator 1α (PGC-1α) (by 21.53- and 18.51-fold), estrogen-related receptor α (ERRα) (by 2.83- and 1.24-fold), and uncoupling protein-1 (UCP-1) (by 1.51- and 0.60-fold) in epididymal adipose tissues. Our results suggest that BAA6 could improve obesity associated with promoting mitochondrial biogenesis and function of adipose tissues in mice.

## 1. Introduction

Obesity is associated with increasing occurrence of metabolic diseases, such as type 2 diabetes [1], as well as cardiovascular and cerebrovascular diseases [2]. Alleviation of obesity can be a means to reduce the risk of metabolic diseases [3]. Currently, the main approaches to treat obesity include diet regulation, exercise, surgical treatment, and drug modulation [4]. Although surgical or medical treatments can significantly reduce body weight, there are many side effects such as portal thrombosis, bleeding, and leak [5,6].

Probiotics intervention can confer great safety and high levels of effectiveness [7]. Given the growing evidence implicating the positive effects of probiotics on obesity treatment, this method is attracting more and more attention [4,8]. Many probiotic strains of the genera *Bifidobacterium* and *Lactobacillus* have anti-obesity effects on different mouse models of obesity [9,10]. *Bifidobacterium animalis* subsp. *lactis* CECT 8145 strain has strong fat-reduction capacity and it modulates lipid metabolism in an obese mouse model [11]. *Lactobacillus acidophilus* NS1 can alleviate HFD-induced obesity in mice by improving lipid metabolism and insulin sensitivity through an AMP-activated protein kinase [12]. *Bifidobacterium animalis* subsp. *lactis* I-2494 was shown to be able to decrease body weight gain and balance gut microbiota composition [13]. It was reported that *Akkermansia muciniphila* (AKK) could decrease the adipose tissue mass and alleviate obesity [14,15]. At present, the mechanisms of probiotics improving obesity are mainly related to fat metabolism, insulin sensitivity, and intestinal microbiota composition. Mitochondria act as the signaling platform to orchestrate integrated physiological responses to maintain health [16]. It is known that mitochondria lie at the center of lipid metabolism, involving processes such as decarboxylation and β-oxidation of fatty acids [17], which are also important sites for producing heat [18]. Some studies found that the reduction of mitochondrial biogenesis and impairment of mitochondrial function can accelerate obesity [19]. There is evidence that the mitochondrial contents and oxidation capacity of fatty acids are lower in obese patients [20]. The gene operon of *Lactobacillus plantarum* may be helpful for mitochondrial fatty acid oxidation [21]. Previous studies showed that estradiol can alleviate obesity through increasing mitochondrial biogenesis and function [22]. However, the relationship between probiotics and mitochondrial biosynthesis and function is less studied in improving obesity.

*Bifidobacterium animalis* subsp. *lactis* A6 (BAA6), a probiotic strain, is isolated from the feces of a centenarian. It was demonstrated that BAA6 has high acid resistance to low pH, which is critical for it surviving through gastric juice [23]. It is unclear whether BAA6 could alleviate obesity and whether this is associated with increasing mitochondrial biogenesis and function of adipose tissues. In this study, we hypothesized that BAA6 could ameliorate obesity related to improving mitochondrial biogenesis and function of adipose tissues.

## 2. Results

### 2.1. BAA6 Accelerated Body Weight and Fat Mass Loss and Changed Serum Lipid Profile Level in Obese Mice

**Figure 1:**
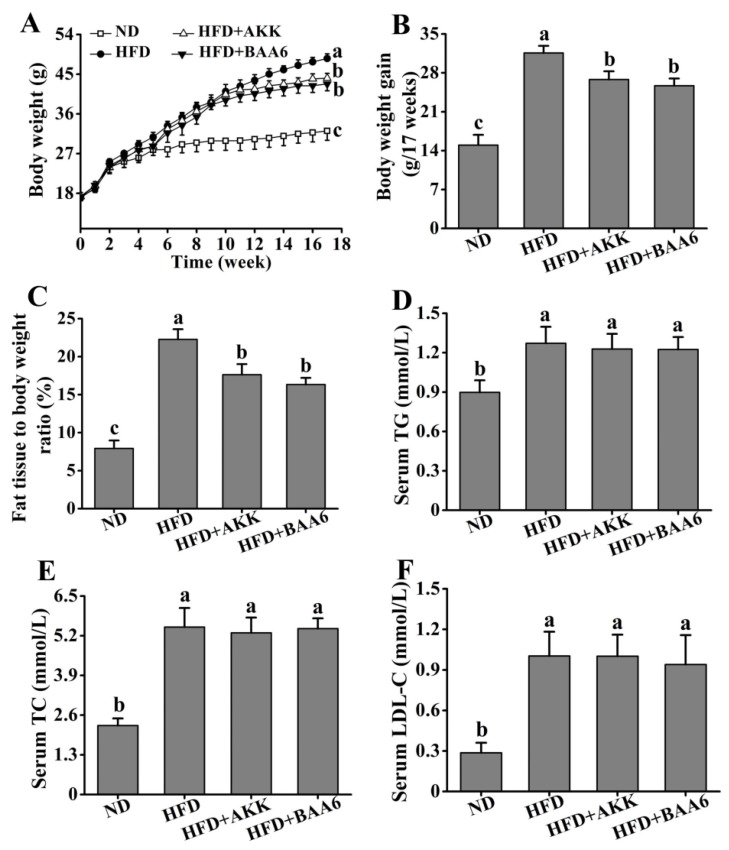
Effects of oral administration of *Bifidobacterium animalis* subsp. *lactis* A6 (BAA6) or *Akkermansia muciniphila* (AKK) on metabolic parameters in high-fat diet (HFD)-fed mice. (**A**) Body weight, (**B**) body weight gain, (**C**) relative adipose tissue, and serum levels of (**D**) total triglyceride (TG), (**E**) total cholesterol (TC), and (**F**) low-density lipoprotein cholesterol (LDL-C) following daily treatment with 10^9^ colony-forming units (CFU)/kg of BAA6 or AKK. Values are expressed as means ± SD (*n* = 6). Bars with different lowercase letters denote significant differences among groups (*p* < 0.05). HFD, fed high-fat diet for 17 weeks; HFD + BAA6, fed high-fat diet and treated with *Bifidobacterium animalis* subsp. *lactis* A6 for the final eight weeks; HFD + AKK, fed high-fat diet and treated with *Akkermansia muciniphila* for the final eight weeks; ND, fed normal diet for 17 weeks.

To understand the effect of BAA6 in ameliorating obesity in mice, we firstly measured food intake of the mice. Compared with the HFD group, there was no significant difference on the weekly food intake of mice after gavage of BAA6 and AKK (*p* > 0.05) (Tables S1–S3, Supplementary Materials). Then, we analyzed the effect of BAA6 treatment on the levels of body weight, fat mass, relative lean weight, and serum lipid profiles of mice. The levels of final body weight, body weight gain, and fat mass were higher in the HFD group than the normal diet (ND) group (*p* < 0.05), which were lower in the HFD + BAA6 and HFD + AKK groups compared with the HFD group (*p* < 0.05) (Figure 1A,B and Figure S1A, Supplementary Materials). Administration of BAA6 and AKK in HFD-fed mice resulted in a lower relative fat weight and higher relative lean weight compared to the HFD group (*p* < 0.05) (Figure 1C and Figure S1B, Supplementary Materials). Meanwhile, the HFD group showed higher levels of serum triglyceride (TG), total cholesterol (TC), and low-density lipoprotein cholesterol (LDL-C) than ND group (*p* < 0.05). The concentrations of serum TG, TC, and LDL-C in the HFD + BAA6 and HFD + AKK groups had no significant difference from those in the HFD group (*p* > 0.05) (Figure 1D–F). Furthermore, we examined the effect of BAA6 on insulin resistance using an intraperitoneal glucose tolerance test (GTT) and insulin tolerance test (ITT). As shown in Figure S2A,B (Supplementary Materials), GTT and ITT were improved in the HFD + BAA6 and HFD + AKK groups. These results suggested that BAA6 treatment could reduce body weight and fat mass.

### 2.2. BAA6 Affected Lipid Metabolism of Obese Mice

**Figure 2:**
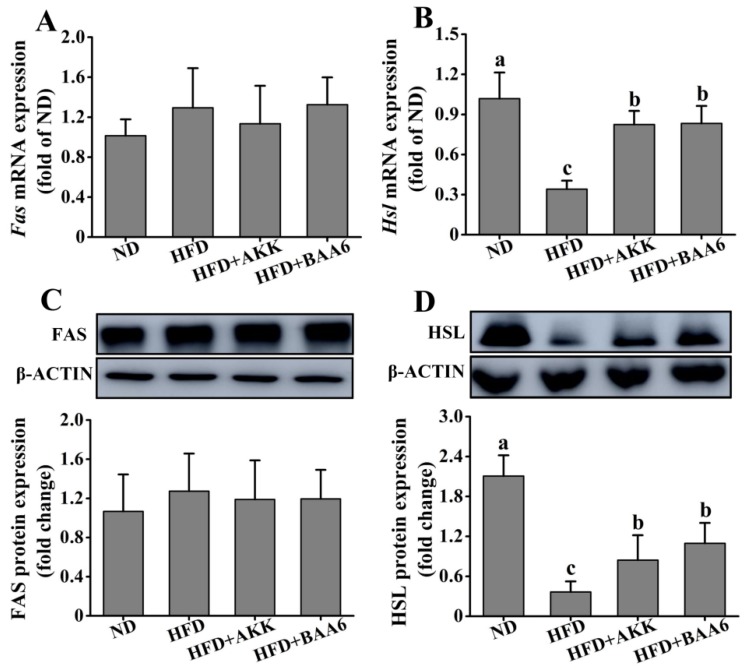
Effects of BAA6 or AKK on lipid metabolism for epididymal adipose tissues in HFD-fed mice. Messenger RNA (mRNA) expression levels of (**A**) fatty acid synthase (*Fas*) and (**B**) hormone-sensitive lipase (*Hsl*), and protein expression levels of (**C**) FAS and (**D**) HSL following daily treatment with 10^9^ CFU/kg BAA6 or AKK. Values are expressed as means ± SD (*n* = 6). Bars with different lowercase letters denote significant differences among groups (*p* < 0.05).

To explore the effect of BAA6 on lipid metabolism, the lipid synthesis-related and catabolism-related genes and proteins in mice were measured. We firstly investigated lipid metabolism in the epididymal adipose tissues. *Fas* messenger RNA (mRNA) and fatty acid synthase (FAS) protein expression levels of epididymal lipid synthesis in the ND group were not significantly different from those in the HFD group (*p* > 0.05). Meanwhile, administration with BAA6 and AKK in HFD mice did not change significantly the *Fas* mRNA and FAS protein expression levels, compared to the HFD group (*p* > 0.05) (Figure 2A,C). The levels of *Hsl* mRNA and hormone-sensitive lipase (HSL) protein expression related to epididymal lipid catabolism are shown in Figure 2B,D. The expression levels of *Hsl* mRNA and HSL protein in epididymal adipose tissue were remarkably downregulated in the HFD group compared with the HFD + BAA6 or HFD + AKK groups (*p* < 0.05). Furthermore, we analyzed lipid metabolism in the hepatic tissues. As shown in Figure S3A–D (Supplementary Materials), the levels of *Fas* mRNA and FAS protein were significantly lower in the ND group than in the HFD group (*p* < 0.05). However, the *Hsl* mRNA and HSL protein expression levels were significantly higher in the ND group than in the HFD group (*p* < 0.05). Compared with the HFD group, there was no significant effect of BAA6 and AKK treatments on the expression of *Fas*, FAS, *Hsl*, and HSL in liver (*p* > 0.05). Overall, these results showed that BAA6 could increase lipid catabolism in epididymal adipose tissues.

### 2.3. BAA6 Modulated Gut Microbiota of Obese Mice

**Figure 3:**
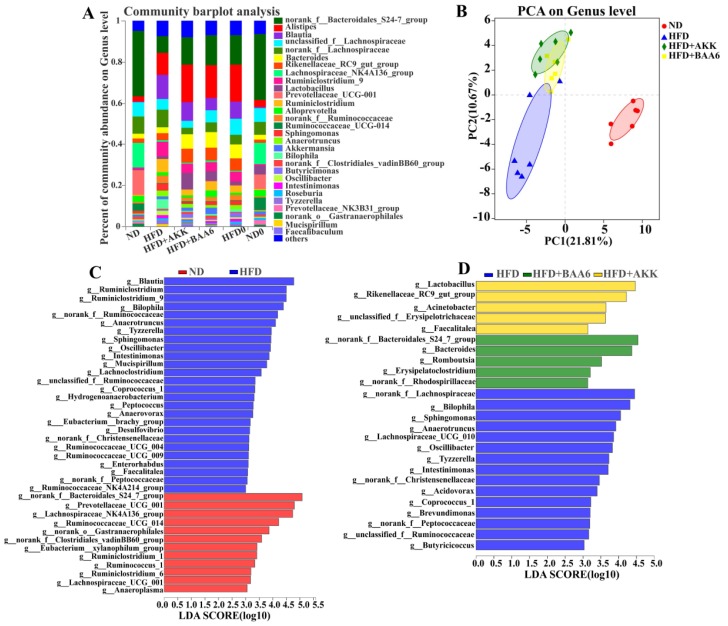
Effects of BAA6 or AKK on gut microbiota in HFD-fed mice. (**A**) Relative abundance of the gut microbial community at the genus level. (**B**) Principal component analysis (PCA) score plots at the genus level. (**C**) Histogram of the linear discriminant analysis (LDA) scores between the ND and HFD groups, and (**D**) histogram of LDA scores among the HFD, HFD + AKK, and HFD + BAA6 groups. The differences with an LDA score greater than three are considered significant. *n* = 6 mice/group. HFD0, fed high-fat diet for nine weeks; ND0, fed normal diet for nine weeks.

The gut microbiota plays a key role in metabolic disorder, especially lipid metabolism [24]. Thus, we wanted to explore the effect of BAA6 on the intestinal microbiota. Firstly, the relative abundance of the gut microbiota was analyzed. At the genus level, *Oscillibacter*, *Bilophila*, *Mucispirillum*, *Anaerotruncus*, *Blautia*, *Sphingomonas*, and *norank_f_Bacteroidates_S24-7_group* in the ND group were found to be differently abundant, compared with the HFD group. Meanwhile, there was a significant reduction in relative abundance of *Oscillibacter* (by 1.86- and 1.94-fold), *Bilophila* (by 3.94- and 5.30-fold), *Anaerotruncus* (by 1.30- and 0.65-fold), and *Sphingomonas* (by 1.01- and 2.55-fold), as well as a significant increase in *norank_f_Bacteroidates_S24-7_group* (by 0.81- and 0.60-fold) and *Lactobacillus* (by 2.63- and 5.01-fold) in the HFD + BAA6 and HFD + AKK groups, when compared with the HFD group (Figure 3A and Figure S4, Supplementary Materials). Furthermore, principal component analysis (PCA) was used to investigate the phylogenetic differences within the gut microbiota (Figure 3B). The HFD group had a distinct microbiota composition that clustered separately from the ND, HFD + BAA6, and HFD + AKK groups with the first principal component (PC1) explaining 21.81% variation and PC2 explaining 10.67% variation (*R^2^* = 0.599; *p* = 0.001). To further analyze the pattern of the microbiota, linear discriminant analysis (LDA) effect size (LEfSe) analysis was employed. Based on the comparison between the ND and HFD groups, 12 key phylotypes were identified as enriched within the ND group, while 26 key phylotypes were identified as enriched within the HFD group such as *Oscillibacter*, *Bilophila, Mucispirillum, Anaerotruncus*, *Sphingomonas*, and *Blautia* (Figure 3C). Furthermore, based on the comparison among HFD, HFD + BAA6, and HFD + AKK groups, five key phylotypes were identified as enriched within the HFD + BAA6 group such as *norank_f_Bacteroidales_S24-7_group*, five key phylotypes were identified as enriched within the HFD + AKK group such as *Lactobacillus*, and 15 key phylotypes were identified as enriched within the HFD group such as *Oscillibacter*, *Bilophila, Anaerotruncus*, and *Sphingomonas* (Figure 3D). Overall, these data strongly demonstrated that BAA6 could reduce the relative abundance of LPS-related strains.

### 2.4. BAA6 Reduced Serum LPS and Inflammatory Cytokines in Fat Tissues of Obese Mice

**Figure 4:**
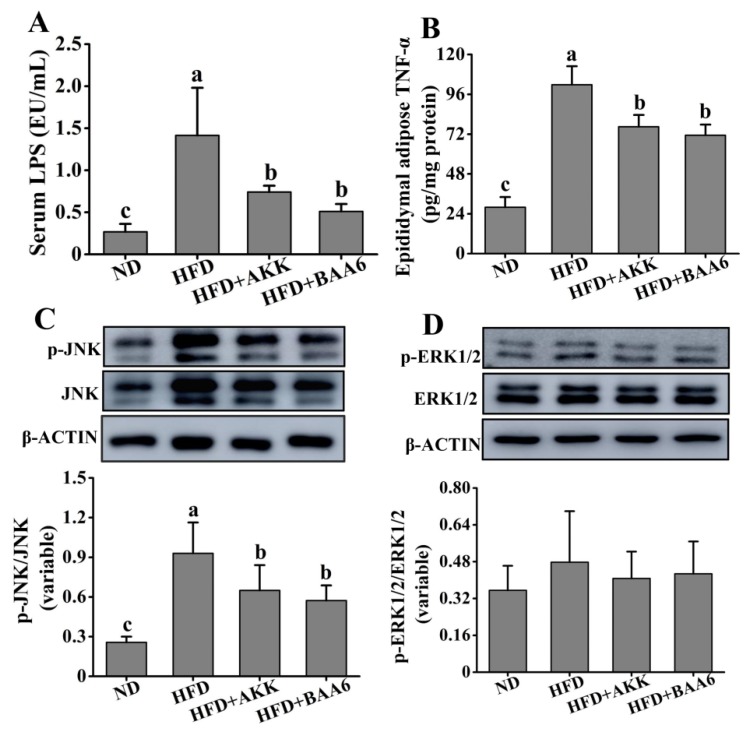
Effects of oral administration of BAA6 or AKK on inflammation in HFD-fed mice. (**A**) The level of serum lipopolysaccharides (LPS). (**B**) Tumor necrosis factor α (TNF-α) concentration in epididymal adipose tissues. Protein expression levels of (**C**) phosphorylated c-Jun N-terminal kinase (*p*-JNK)/JNK and (**D**) phosphorylated extracellular signal-regulated kinase 1/2 (*p*-ERK1/2)/ERK1/2 in epididymal fat tissues following daily treatment with 10^9^ CFU/kg BAA6 or AKK. Values are expressed as means ± SD (*n* = 6). Bars with different lowercase letters denote significant differences among groups (*p* < 0.05).

Obesity is associated with the state of chronic inflammation and abnormal production of proinflammatory mediators, such as tumor necrosis factor α (TNF-α). We confirmed the efficacy of BAA6 in decreasing the LPS-producing microbiota. Evidence strongly suggests that the overgrowth of LPS-related strains in the gut is closely related to the increase in serum LPS. LPS is a strong stimulator for the release of TNF-α [25]. Thus, we wanted to explore the relationship between LPS and TNF-α. The concentration of serum LPS was firstly explored. Mice in the HFD group exhibited an elevated level of serum LPS, which was lower in the ND group (*p* < 0.05). Compared to the HFD group, the serum LPS concentration was remarkably decreased in the HFD + BAA6 and HFD + AKK groups (*p* < 0.05) (Figure 4A). Then, we explored the TNF-α concentration in the epididymal adipose tissues. As shown in Figure 4B, the TNF-α concentration in the HFD group was remarkably increased, compared with the ND group (*p* < 0.05). However, administration with BAA6 and AKK significantly decreased the TNF-α level (*p* < 0.05). We found a change in serum LPS level consistent with the change in TNF-α concentration in the epididymal fat tissues. To further investigate how LPS could induce the change in TNF-α, the levels of phosphorylation of c-Jun N-terminal kinase (JNK) and extracellular signal-regulated kinase 1/2 (ERK1/2) were explored. As shown in Figure 4C, the HFD group had a higher phosphorylated (*p*)-JNK/JNK ratio compared to the ND group (*p* < 0.05). Furthermore, BAA6 and AKK supplementation significantly decreased the *p*-JNK/JNK proportion (*p* < 0.05). The ratio of *p*-ERK1/2/ERK1/2 in the HFD group was not significantly different from the ND group (*p* > 0.05). Meanwhile, the levels of *p*-ERK1/2/ERK1/2 in the HFD + BAA6 and HFD + AKK groups had no significant difference from those in the HFD group (*p* > 0.05) (Figure 4D). These data indicated that BAA6 could decrease TNF-α expression in epididymal fat, which was likely to be associated with inhibiting the JNK signaling pathway through reducing serum LPS level.

### 2.5. BAA6 Increased Endothelial Nitric Oxide Synthase (eNOS) Expression and Mitochondrial Biogenesis in Fat Tissues of Obese Mice

**Figure 5:**
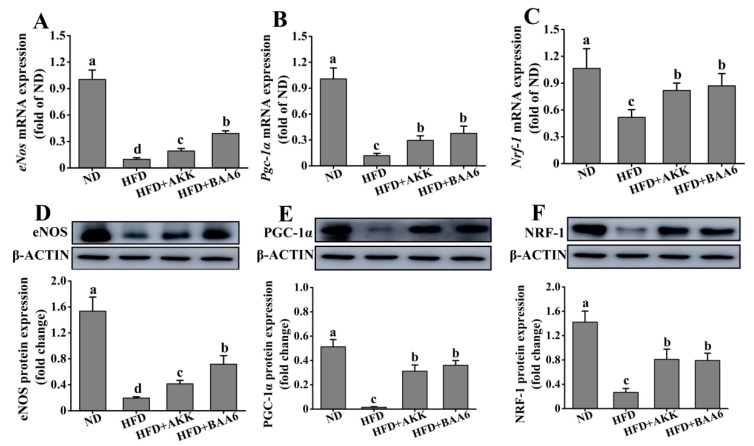
Effects of oral administration of BAA6 or AKK on endothelial nitric oxide synthase (eNOS) expression and mitochondrial biogenesis of epididymal adipose tissues in HFD-fed mice. (**A**) mRNA expression levels of endothelial nitric oxide synthase (*eNos*), (**B**) peroxisome proliferator-activated receptor γ coactivator 1α (*Pgc-1α*), and (**C**) nuclear respiratory factor-1 (*Nrf-1*) and protein expression levels of (**D**) eNOS, (E) PGC-1α, and (**F**) NRF-1 following daily treatment with 10^9^ CFU/kg BAA6 or AKK. Values are expressed as means ± SD (*n* = 6). Bars with different lowercase letters denote significant differences among groups (*p* < 0.05).

TNF-α could regulate eNOS expression [26], and several studies demonstrated that eNOS could promote mitochondrial biogenesis [27]. Moreover, mitochondria are the main site of catabolism. Therefore, we investigated the effect of BAA6 on eNOS expression and mitochondrial biosynthesis in epididymal fat tissues. The levels of *eNos* mRNA and eNOS protein expression were firstly measured in epididymal adipose. The levels of *eNos* mRNA and eNOS protein expression in the HFD group were lower than those in the ND group (*p* < 0.05). After adding BAA6 and AKK, the magnitudes of *eNos* mRNA and eNOS protein expression were markedly increased compared with the HFD group (*p* < 0.05) (Figure 5A,D). eNOS is involved in the activation of peroxisome proliferator-activated receptor γ coactivator 1α (PGC-1α) expression. PGC-1α is a master regulator of cellular metabolism, and it controls the expression of mitochondrial genes. Next, we investigated *Pgc-1α* mRNA and PGC-1α protein expression in epididymal fat of obese mice. As shown in Figure 5B,E, the levels of *Pgc-1α* mRNA and PGC-1α protein expression were lower in the HFD group than in the ND group (*p* < 0.05). Compared with the HFD group, *Pgc-1α* mRNA and PGC-1α protein expression was significantly increased in the HFD + BAA6 and HFD + AKK groups (*p* < 0.05). Nuclear respiratory factor-1 (NRF-1) was identified as an important target for the induction of mitochondrial biogenesis by PGC-1α. Thus, we further explored the levels of *Nrf-1* mRNA and NRF-1 protein expression. The levels of *Nrf-1* mRNA and NRF-1 protein expression were lower in the HFD group compared to the ND group (*p* < 0.05). After administration of BAA6 and AKK, *Nrf-1* mRNA and NRF-1 protein expression levels were significantly increased compared to the HFD group (*p* < 0.05) (Figure 5C,F). Overall, these results showed that BAA6 increased mitochondrial biogenesis, which was associated with increased eNOS expression.

### 2.6. BAA6 Reinforced Mitochondrial Function in Fat Tissues of Obese Mice

**Figure 6:**
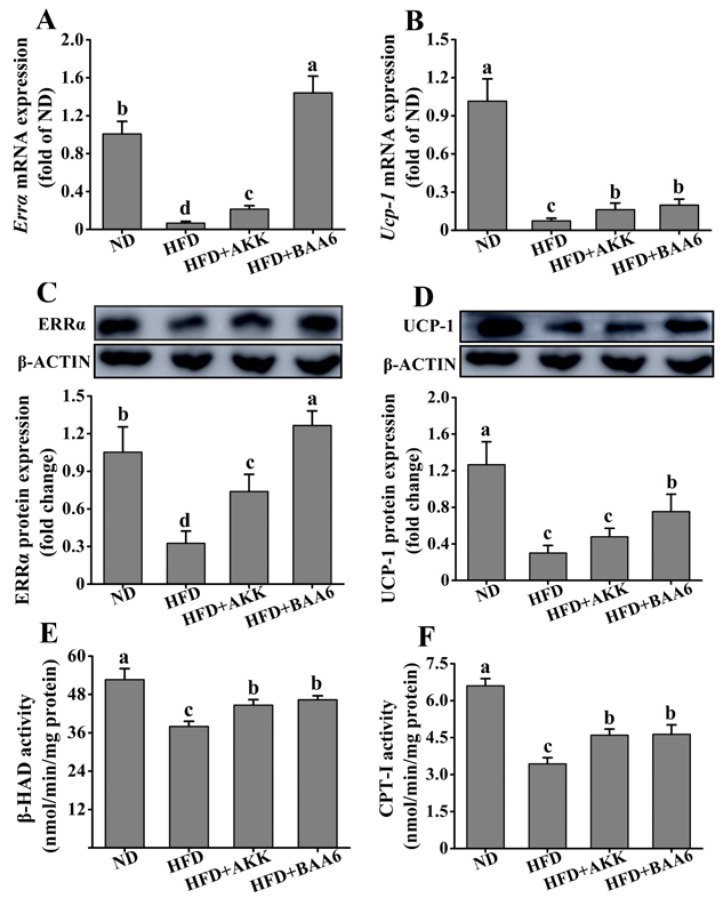
Effects of oral administration of BAA6 or AKK on mitochondrial function of epididymal fat tissues in HFD-fed mice. mRNA expression levels of (**A**) estrogen-related receptor α (*Errα*) and (**B**) uncoupling protein-1 (*Ucp-1*), protein expression levels of (**C**) ERRα and (**D**) UCP-1, and enzyme activities of (**E**) β-hydroxyacyl CoA dehydrogenase (β-HAD) and (**F**) carnitine palmitoyl transferase I (CPT-I) following daily treatment with 10^9^ CFU/kg of BAA6 or AKK. Values are expressed as means ± SD (*n* = 6). Bars with different lowercase letters denote significant differences among groups (*p* < 0.05).

Next, we examined the effect of BAA6 on the mitochondrial function of epididymal fat tissues. The β-oxidation and thermogenic function of mitochondria were studied. Estrogen-related receptor α (ERRα) acts as a regulator of β-oxidation via its control of the medium-chain acyl-coenzyme A dehydrogenase promoter. We firstly examined *Errα* mRNA and ERRα protein levels in epididymal fat. The HFD group showed a striking reduction in *Errα* mRNA and ERRα protein levels, when compared with the ND group (*p* < 0.05). However, *Errα* mRNA and ERRα protein levels were significantly increased in the HFD + AKK and HFD + BAA6 groups compared to the HFD group (*p* < 0.05) (Figure 6A,C). Then, we explored β-hydroxyacyl CoA dehydrogenase (β-HAD) and carnitine palmitoyl transferase I (CPT-I) enzyme activities, which are involved in the regulation of β-oxidation. The β-HAD and CPT-I activities were lower in the HFD group compared to the ND group (*p* < 0.05). After administration with BAA6 and AKK, the β-HAD and CPT-I activities were significantly increased compared to the HFD group (*p* < 0.05) (Figure 6E,F). Furthermore, an elevation in mitochondrial uncoupling could dissipate energy as heat and contribute to an increase in fatty acid utilization. Thus, we investigated uncoupling protein-1 (*Ucp-1*) mRNA and UCP-1 protein expression in epididymal fat tissues. Both *Ucp-1* mRNA and UCP-1 protein levels were lower in the HFD group than in the ND group (*p* < 0.05). Mice in the HFD + BAA6 group possessed a more potent effect on upregulating *Ucp-1* mRNA and UCP-1 protein compared with the HFD group (*p* < 0.05). Administration of AKK in the HFD group resulted in significantly higher *Ucp-1* mRNA expression compared to the HFD group (*p* < 0.05). Although there was no significant difference in UCP-1 protein level (*p* > 0.05), the trend was similar to mRNA expression (Figure 6B,D). Altogether, these data indicated that BAA6 could increase mitochondrial function.

## 3. Discussion

This study demonstrated the effect of oral administration with probiotic BAA6 on improving obesity. The results showed that supplementation with BAA6 significantly reduced body weight and relative adipose weight, while it increased lipolysis metabolism in adipose tissues, indicating that BAA6 has the potential to improve obesity. Meanwhile, BAA6 showed a better effect on improving obesity than AKK, which was reported to alleviate obesity in previous studies [28]. Previous studies showed that obesity-resistant mice, fed with HFD, had lower body weight and fat gain than obesity-prone mice. Nevertheless, serum lipid profiles in obesity-resistant mice had no remarkable difference from obesity-prone mice during high-fat feeding [29]. Our results also found that there was no significant difference among the HFD, HFD + BAA6, and HFD + AKK groups in terms of serum TG, TC, and LDL-C levels. This phenomenon may be due to the long-term HFD treatment, which maintains serum TG, TC, and LDL-C at a higher level. Although there was intervention due to probiotics like BAA6 and AKK, serum TG, TC, and LDL-C levels were not dramatically reduced in a short time.

The imbalance of the gut microbiota plays an important role in the development of obesity, and obesity is also associated with a chronic inflammatory state [24]. It is now known that inflammation-related obesity is linked to LPS [30]. Song et al. found that HFD treatment could induce higher levels of gut LPS-producing bacteria, especially *Bilophila* and *Oscillibacter* [31]. Meanwhile, emerging evidence suggested that the overgrowth of *Mucispirillum* strains in gut was positively correlated with LPS [32]. Previous clinical and animal experiments confirmed that probiotics could alleviate obesity and improve the composition of gut microbiota [33]. Consistent with these studies, BAA6 intervention significantly decreased the relative abundance of LPS-related bacteria, such as *Bilophila* and *Oscillibacteron*. Supplementation with AKK could increase the levels of β-sitosterol in the gut [34], and β-sitosterol had positive effects on the growth of *Lactobacillus* [35]. Thus, this may be the reason that AKK could markedly increase the relative abundance of *Lactobacillus* in our study. It was reported that *Lactobacillus* could effectively suppress body weight gain [36] and had anti-inflammatory effects [37]. Hence, the positive effects of AKK may be related to *Lactobacillus.* Moreover, we found that BAA6 could dramatically increase the relative abundance of *Norank_f_Bacteroidales_S24-7_group*. Some studies found that *Bifidobacterium* could increase the relative abundance of *Norank_f_Bacteroidales_S24-7_group* [38], which was similar to our findings. The *norank_f_Bacteroidales_S24-7_group* might exert beneficial effects against obesity due to alteration of the gut microbiota [39]. It was also involved in host–microbe interactions which affected gut function and health [40]. When mice were fed with a low-fat diet, the *norank_f_Bacteroidales_S24-7_group* was abundant [41]. In addition, some research found that the *norank_f_Bacteroidales_S24-7_group* was strongly correlated with the suppression of inflammatory markers in obese mice [42]. Our data showed that probiotic BAA6 treatment remarkably increased the relative abundance of the *norank_f_Bacteroidales_S24-7_group*.

Many evidence strongly suggested that the overgrowth of LPS-related strains in gut was closely related to the increase in serum LPS [43]. The leakage of LPS to circulation was based on the high permeability of the intestine [25]. Many studies showed that the mucus layer’s integrity had a positive effect on maintaining intestinal permeability. It was shown that the thickness of the mucus layer was decreased, and intestinal permeability was increased in obese mice, particularly due to the increased population of harmful microorganisms [44]. We found that the level of serum LPS was higher in the HFD group than in the ND group, which might be due to the lower mucus layer thickness and higher relative abundance of LPS-related bacteria such as *Bilophila*, *Oscillibacteron*, and *Mucispirillum*. Several strategies, such as the utilization of probiotics, were suggested to be able to regulate gut microbiome dysbiosis by increasing the population of beneficial bacteria while reducing the growth of indigenous pathobionts and opportunistic pathogens [45,46]. Cani et al. reported that HFD was associated with lower *Bifidobacterium* species in the gut of mice [25]. Probiotics exerted beneficial effects on the host by improving intestinal microbial balance and colonization [47]. They might be involved in the stimulation of mucin secretion and reduction of enterocyte apoptosis to improve intestinal permeability [48,49]. *Faecalibacterium prausnitzii* [49] and AKK [50] were negatively correlated with LPS in obesity. Some studies indicated that probiotics could promote the secretion of mucus to reduce intestinal permeability [51]. We observed that administration of BAA6 and AKK in HFD mice decreased serum LPS, which might be due to the higher mucus layer thickness and lower relative abundance of LPS-related bacteria such as *Bilophila* and *Oscillibacter*.

A better understanding of the cross-talk among inflammation, adipose tissue, and metabolism energy is crucial for elucidating the underlying mechanism of obesity-related diseases. Moreover, the adipose tissues are major sites for the storage of excess energy and inflammatory response reactions [52]. It is not clear why low-grade inflammation exists, but recent advances revealed a mechanism related to the leakage of LPS from gut [25]. A constantly elevated serum LPS was considered to cause inflammation, dyslipidemia, and metabolic disease [53]. Furthermore, obesity is associated with abnormal TNF-α production of adipose tissues [53]. LPS is a strong stimulator for the release of several cytokines like TNF-α, implying its critical role in driving the inflammatory potential of adipocytes [25]. It could induce the production of TNF-α by activating JNK pathways [54]. Our study revealed that the *p*-JNK/JNK ratio in the HFD + BAA6 and HFD + AKK groups was significantly decreased compared to that in the HFD group. We also found that the concentrations of TNF-α in epididymal fat in the HFD + BAA6 and HFD + AKK groups were lower than those in the HFD group, which was likely associated with the regulation of serum LPS by the JNK signaling pathway. TNF-α could be overproduced in the adipose tissues of the obese model, playing an important role in this process. There was a three-fold increase of TNF-α levels in obese individuals [55,56]. Furthermore, the higher level of TNF-α could lead to the downregulation of eNOS [26]. Lower expression of eNOS in adipose tissues is an important risk factor for obesity [51]. Our results also showed significantly lower levels of *eNos* mRNA and eNOS protein in the HFD group. However, supplementation with BAA6 and AKK in HFD mice could induce *eNos* mRNA and eNOS protein expression.

As the important site of energy metabolism, defective metabolism of the mitochondria was recently suggested as a major cause of obesity [51]. It was reported that lipid metabolism could be improved by enhancing mitochondrial biogenesis and function [57,58]. Therefore, mitochondrial biogenesis and function are pivotal to mitigate obesity. Several studies demonstrated that eNOS was involved in mitochondrial signaling pathways [59] and promoted mitochondrial biogenesis [27]. eNOS is also involved in the activation of PGC-1α, which is the master regulator of cellular metabolism [60] and controller of mitochondrial biogenesis [61]. Moreover, the content of lipid was noticeably diminished by increasing mitochondrial biogenesis and function [62]. Interestingly, mitochondria in mice lacking the gene encoding endothelial NO synthase were somewhat less densely packed compared to wild-type mice [27]. These changes were accompanied by a reduction of PGC-1α, indicating that basal mitochondrial contents were affected by the loss of eNOS. Our study showed that *Pgc-1α* gene and PGC-1α protein levels in epididymal adipose tissues were reduced in the HFD group and markedly elevated by treatments with probiotics BAA6 or AKK in HFD-fed mice. These results indicated that mitochondrial biogenesis and metabolism of epididymal adipose tissues in HFD mice could be improved by BAA6 or AKK treatment. Meanwhile, PGC-1α could in turn activate the expression of NRF-1 and ERRα [63]. NRF-1 could regulate mitochondrial DNA copy numbers and transcriptional activities [64]. The associated increase in NRF-1 expression coincided with elevated expression of cytochrome c and enhanced mitochondrial densities [65]; thus, NRF-1 could increase the oxidative respiration of mitochondria. ERRα is the vital step in mitochondrial fatty acid β-oxidation, and it is considered to be one of the gatekeepers that control the fatty acid β-oxidation rate in the cell [66]. We observed that addition of probiotics BAA6 and AKK in HFD-fed mice could markedly improve *Nrf-1* and *Errα* mRNA, as well as NRF-1 and ERRα protein expression of epididymal adipose tissues. These findings indicated that mitochondrial fatty acid β-oxidation in the epididymal adipose tissues of HFD mice could be significantly improved by BAA6 or AKK treatment. Some researchers found that CPT-I and β-HAD were the key regulatory enzyme in mitochondrial β-oxidation [67,68]. Our study also found that the β-HAD and CPT-I activities were significantly increased by treatments with probiotics BAA6 or AKK in HFD-fed mice, which meant that the effect of mitochondria on fatty acid β-oxidation was increased by BAA6 or AKK treatment. UCP-1 is an inner mitochondrial membrane protein, which may also be induced by PGC-1α [69]. Previous studies showed that UCP-1 could uncouple mitochondrial oxidative phosphorylation to produce heat instead of ATP synthesis [18]. The levels of *Ucp-1* mRNA and UCP-1 protein in the epididymal adipose tissues of HFD mice were significantly lower than HFD-fed mice administrated with BAA6, which meant that the thermogenic function of mitochondria in the epididymal adipose tissues of HFD mice was improved by BAA6 treatment. Furthermore, the upregulated expression of UCP-1 in adipose tissue could boost heat production, which had positive effects on alleviating obesity [70]. These findings indicate that the probiotic BAA6 has strong potential to promote mitochondrial biogenesis and function to accelerate lipid metabolism, which is similar to the findings by Lei et al. [57] and Tsutsumi et al. [58].

In conclusion, our study demonstrated the beneficial effect of probiotic BAA6 on the alleviation of obesity. We also provided new insight into the relationship between BAA6 and mitochondrial biosynthesis and function of adipose tissues to improve obesity.

## 4. Materials and Methods

### 4.1. Preparation of Bacterial Cultures

BAA6 (CGMCC No. 9273) was isolated from the feces of a centenarian in Bama, Guangxi, China. It was grown anaerobically (100% N_2_) at 37 °C in a basal liquid medium that contained the following (per liter of deionized water): 10 g of beef extract, 5 g of yeast extract, 10 g of tryptone peptone, 20 g of glucose, 2 g of triamine citrate, 2 g of K_2_HPO_4_, 0.5 g of MgSO_4_, 5 g of sodium acetate, 0.5 g of L-cysteine, 0.25 g of MnSO_4_, and 1 mL of Tween-80. The solid medium for BAA6 was based on liquid medium plus agar (15 g, per liter liquid medium).

AKK (JCM 30893) was purchased from the Japan Collection of Microorganisms RIKEN BioResource Research Center, Ibaraki, Japan. It was grown anaerobically (H_2_:CO_2_:N_2_, 1:1:8) at 37 °C in nutrient broth (5 g of peptone and 3 g of beef extract, per liter of deionized water). The solid medium for AKK was Columbia blood agar with 5% horse blood.

The bacteria were cultivated overnight and harvested by centrifuging at 3000× g for 15 min at 4 °C, washed twice with 0.9% saline solution, and resuspended in 0.9% saline solution. Bacterial concentration was determined by plate count, which was carried out in the respective growth conditions and above-mentioned solid media.

### 4.2. Animals and Diets

All animal studies were approved by the Animal Experimentation Ethics Committee of the China Agricultural University (Beijing, China). Male C57BL/6 mice (*n* = 26, four weeks old) were purchased from Beijing HFK Bioscience Co. Ltd. (Beijing, China). All animals were acclimated for one week under the following conditions: room temperature 23 ± 1 °C; humidity 50% ± 5%; 12-h light/dark cycle. During this period, food and water were provided ad libitum. At the end of the first week, six mice were continually fed with a ND to serve as a control group (ND group, 10% energy from fat), while the other 20 mice were placed on an HFD (60% energy from fat) for nine weeks to induce obesity [16]. After a nine-week feeding period with the HFD, the body weights of 18 mice were 20% more than the mean body weight of mice in the ND group, which suggested that the obesity model was successfully established [71]. Then, they were randomly divided into (1) HFD group (*n* = 6, fed with HFD for the final eight weeks), (2) HFD + BAA6 group (*n* = 6, fed with HFD and treated with BAA6 for the final eight weeks (10^9^ CFU/kg per day)), (3) HFD + AKK group (*n* = 6, fed with HFD and treated with AKK for the final eight weeks (10^9^ CFU/kg per day) as positive control). AKK could alleviate obesity [14], which was positively correlated with anti-inflammation [72], as well as the levels of fat browning, fatty acid oxidation, and heat production [73]. BAA6 and AKK were suspended in 0.9% saline solution, then administrated by oral gavage daily from the 10th week. Mice in ND and HFD groups were given 0.9% saline solution only. ND and HFD (Table S4, Supplementary Materials) were obtained from Research Diets Inc., New Brunswick, NJ, USA. Body weight and food intake were recorded every week. Mice were fasted overnight then anesthetized with Zoletil^®^ and Rompun^®^. They were sacrificed by cervical dislocation after blood collection. Serum, adipose tissue, and liver tissue were stored at −80 °C for further analyses.

### 4.3. Body Fat Analysis

Body fat was determined with the nuclear magnetic resonance system using a Body Composition Analyzer MiniQMR23-060H-I (Shanghai Niumag Corporation, Shanghai, China). It was measured in live conscious mice with ad libitum access to diet, as described previously [74].

### 4.4. Lipid Profile Analysis

Serum levels of TG, TC, and LDL-C were measured by the certified core clinical laboratory at the No. 3 Hospital of Beijing University, who used the respective commercial kits and Chemistry Analyzer (BS-350E) provided by Shenzhen Mindray Biomedical Electronics Co., Ltd. (Shenzhen, China) [75].

### 4.5. Glucose Tolerance Test and Insulin Tolerance Test

For the glucose tolerance test, mice were intraperitoneally injected with glucose at 2 g per kg body weight after 12 h of fasting. For the insulin tolerance test, mice were intraperitoneally injected with insulin at 1 IU per kg body weight (Eli Lilly and Co., Humalog Insulin) after 6 h of fasting. In all tests, tail blood glucose levels were measured with a glucometer (TheraSense Freestyle) at the indicated times (0, 15, 30, 60, 90, and 120 min) after injection.

### 4.6. PCR Amplification and Sequencing Analysis of Feces

Feces bacterial analysis was carried out as described previously [76]. Briefly, total DNA was extracted from the fecal samples using the Feces DNA Extraction kit (BioTeKe, Beijing, China). Fecal DNA samples were used as the template for PCR amplification of the V3–V4 hyper-variable regions of 16S ribosomal RNA (rRNA) genes by using the primers 338F (5′–GTGCCAGCMGCCGCGG–3′) and 806R (5′–CCGTCAATTCMTTTRAGTTT–3′). PCR amplification was performed on an ABI GeneAmp^®^9700 PCR System (Applied Biosystems, Foster, CA, USA). Sequencing of the PCR amplification products was performed on an Illumina Miseq platform at Majorbio Bio-Pharm Technology (Shanghai, China).

The processing and bioinformatics analyses of the raw data were performed as described previously [77]. A 97% similarity cutoff was applied to cluster operational taxonomic units (OTUs) with UPARSE (version 7.1 http://drive5.com/uparse/), and chimeric sequences were identified and removed with UCHIME. The taxonomy of each 16S rRNA gene sequence was analyzed using the RDP Classifier (http://rdp.cme.msu.edu/) against the SILVA (SSU115) 16S rRNA database. Analysis of the composition of microbiomes (ANCOM) was carried out in the software R (version 3.6.1). Furthermore, PCA was performed using the R package. The LEfSe analysis (http://huttenhower.sph.harvard.edu/lefse/) was performed to identify the highly dimensional gut microbes with a threshold of 3.0 on the LDA score for discriminative features. Differences were considered to be significant at *p* < 0.05.

### 4.7. Determination of Endotoxin

Serum LPS was assessed using a quantitative Chromogenic End-point Tachypleus Amebocyte Lysate assay kit (Xiamen Houshiji, China) as previously described [78].

### 4.8. Determination of TNF-α Level in Epididymal Adipose Tissues

TNF-α level in epididymal adipose tissues was determined using the Mouse TNF-α ELISA Kit (ExCell Biology, Shanghai, China), according to the manufacturer’s instruction [79]. The protein concentrations were measured by Pierce™ BCA Protein Assay Kit (Thermo Fisher Scientific, Rockford, MI, USA).

### 4.9. Activity Assay for β-Hydroxyacyl CoA Dehydrogenase and Carnitine Palmitoyl Transferase I

β-HAD activity was assayed in whole lysates prepared from frozen epididymal adipose tissues as described previously [80]. Firstly, 40 μL of homogenate (1 mg/mL) was added to a cuvette, which was then was brought to a final volume of 670 μL with 800 μL of 50 mmol/L imidazole (pH 7.4) and 10 μL of 1.5 mmol/L reduced nicotinamide adenine dinucleotide (NADH). The reaction was initiated by the addition of 10 μL of 2 mmol/L acetoacetyl-CoA. Absorbance at 340-nm wavelength was followed for 5 min with a spectrophotometer. CPT-I activity was assayed in whole lysates prepared from frozen epididymal adipose tissues as described previously [81].

### 4.10. Quantitative RT-PCR of mRNA Expression

Analysis of mRNA levels was performed as previously described [52]. Briefly, total RNA was isolated from epididymal adipose, hepatic tissues, and ileum using TRIzol (Invitrogen, Carlsbad, CA, USA). Complementary DNA (cDNA) was prepared by reverse transcription of 2 mg of total RNA using a first-strand cDNA synthesis kit (Takara Biotechnology, Dalian, China). RT-PCR was performed using LightCycler^®^ 96 (Roche, Mannheim, Germany). The sequences of primers are listed in Table S5 (Supplementary Materials).

### 4.11. Immunoblot Analysis

Epididymal fat and hepatic tissues were lysed in ice-cold condition with lysis buffer, and proteins were separated by protein gel electrophoresis, followed by transfer to a polyvinylidene difluoride membrane (Millipore, Boston, MA, USA). Later individual immunoblots were probed with antibodies. Finally, the band signal intensities were determined by Image J software [82]. The following primary antibodies were used: FAS, HSL, ERK1/2, *p*-ERK1/2, JNK, *p*-JNK, and eNOS antibodies (Cell Signaling Technology, Boston, MA, USA). PGC-1α, NRF-1, ERRα, and UCP-1 antibodies were from Abcam (Cambridge, UK). β-Actin antibody was purchased from Bioss (Beijing, China).

### 4.12. Statistical Analysis

Data were expressed as means ± standard deviations (SD). Data analysis was carried out using SPSS software (version 17.0 SPSS Inc., Chicago, IL, USA). Before performing one-factor ANOVA analysis, Levene’s test was used to test for equal variance. After one-factor ANOVA, Duncan’s post hoc test was used to evaluate differences between groups (*p* < 0.05).

## Supplementary Materials

The following are available online. Figure S1: Effects of oral administration of BAA6 or AKK on fat mass and relative lean weight in HFD-fed mice; Figure S2: Enhanced inhibition effects of BAA6 or AKK on insulin resistance in HFD-fed mice; Figure S3: Effects of BAA6 or AKK on the lipid metabolism for liver in HFD-fed mice; Figure S4: Effects of BAA6 or AKK on the percentage of relative abundance for gut microbiota at the genus level in HFD-fed mice; Table S1: Effects of oral administration of BAA6 or AKK on food intake in HFD-fed mice; Table S2: Effects of oral administration of BAA6 or AKK on vitamin intake in HFD-fed mice; Table S3: Effects of oral administration of BAA6 or AKK on mineral intake in HFD-fed mice; Table S4: Composition of diets; Table S5: The sequences of primers used in epididymal adipose and liver tissues gene expression.
