# Supplementary material for: Bifidobacterium animalis subsp. lactis A6 Alleviates Obesity Associated with Promoting Mitochondrial Biogenesis and Function of Adipose Tissue in Mice
Source: Molecules. 2020 Mar 25;25(7):1490. doi: 10.3390/molecules25071490 (PMC7180933; doi:10.3390/molecules25071490)
Supplement: Supplementary file 1 [file molecules-25-01490-s001.zip › Supplementary/Supplementary data-figure S1-S2-S3-S4.doc]

**Supplementary data：**

**Figure. S1** Effects of oral administration of BAA6 or AKK on fat mass and relative lean weight in HFD-fed mice. (**A**) Fat mass and (**B**) relative lean weight during 10^9^ CFU/kg of BAA6 or AKK daily treatment. Values are expressed as means ± SD (n = 6, respectively). Bars with different lowercase letters denote significant differences among groups (*p* < 0.05). HFD, fed high fat diet for 17 weeks; HFD+BAA6, fed high fat diet and treated with *Bifidobacterium animalis subsp. Lactis* A6 for the final 8 weeks; HFD+AKK, fed high fat diet and treated with *Akkermansia muciniphila* for the final 8 weeks; ND, fed normal diet for 17 weeks.

**Figure. S2** Enhanced inhibition effects of BAA6 or AKK on insulin resistance in HFD-fed mice. (**A**) Glucose tolerance test (GTT) and (**B**) Insulin tolerance test (ITT) of HFD-fed mice. Values are expressed as means ± SD (n = 6, respectively). **p* < 0.05 vs. ND group; # *p* < 0.05 vs. HFD group.

**Figure. S3** Effects of BAA6 or AKK on the lipid metabolism for liver in HFD-fed mice. mRNA expression levels of (**A**) fatty acid synthase (*Fas*) and (**B**) hormone sensitive lipase (*Hsl*), and protein expression levels of (**C**) FAS and (**D**) HSL during 10^9^ CFU/kg of BAA6 or AKK daily treatment. Values are expressed as means ± SD (n = 6, respectively). Bars with different lowercase letters denote significant differences among groups (*p* < 0.05).

**Figure. S****4** Effects of BAA6 or AKK on the percentage of relative abundance for gut microbiota at the genus level in HFD-fed mice. Values are expressed as means ± SD (n = 6, respectively). These differences were significant as assessed by ANCOM. ^*^*p* < 0.05 vs. ND group. ^#^*p* < 0.05 vs. HFD group. ANCOM, analysis of composition of microbiomes.
