# Supplementary material for: Bifidobacterium animalis subsp. lactis A6 Alleviates Obesity Associated with Promoting Mitochondrial Biogenesis and Function of Adipose Tissue in Mice
Source: Molecules. 2020 Mar 25;25(7):1490. doi: 10.3390/molecules25071490 (PMC7180933; doi:10.3390/molecules25071490)
Supplement: Supplementary file 1 [file molecules-25-01490-s001.zip › Supplementary/Supplementaty Table.doc]

Supplementary Table S1. Effects of oral administration of BAA6 or AKK on food intake in HFD-fed mice^1^.

|  | Food intake, g | | | |
| --- | --- | --- | --- | --- |
| Time, weeks | ND | HFD | HFD+AKK | HFD+BAA6 |
| 1 | 18.78 ± 2.33^a^ | 14.86 ± 1.93^b^ | 14.82 ± 2.78^b^ | 14.98 ± 1.97^b^ |
| 2 | 20.51 ± 2.90^a^ | 17.90 ± 2.18^ab^ | 16.79 ± 1.83^b^ | 17.35 ± 2.48^ab^ |
| 3 | 21.53 ± 2.01^a^ | 18.40 ± 1.45^b^ | 18.41 ± 1.14^b^ | 16.85 ± 1.61^b^ |
| 4 | 20.41 ± 2.34^a^ | 18.53 ± 1.11^a^ | 18.75 ± 0.89^a^ | 18.89 ± 2.37^a^ |
| 5 | 21.00 ± 1.71^a^ | 19.73 ± 2.71^a^ | 19.18 ± 2.84^a^ | 18.55 ± 2.00^a^ |
| 6 | 21.09 ± 1.43^a^ | 19.48 ± 2.93^a^ | 18.32 ± 2.47^a^ | 18.77 ± 2.90^a^ |
| 7 | 21.14 ± 1.84^a^ | 18.94 ± 1.20^a^ | 19.24 ± 2.16^a^ | 19.06 ± 1.58^a^ |
| 8 | 21.27 ± 1.72^a^ | 18.18 ± 1.01^b^ | 19.62 ± 2.63^ab^ | 18.92 ± 1.48^ab^ |
| 9 | 21.09 ± 1.74^a^ | 19.25 ± 3.02^a^ | 18.78 ± 2.54^a^ | 18.55 ± 1.45^a^ |
| 10 | 20.92 ± 1.21^a^ | 18.61± 2.31^a^ | 18.71 ± 2.57^a^ | 18.62 ± 3.13^a^ |
| 11 | 21.65 ± 1.26^a^ | 18.32± 1.35^b^ | 18.80 ± 2.76^b^ | 18.81± 2.23^b^ |
| 12 | 21.84 ± 0.63^a^ | 18.81 ± 4.33^a^ | 18.41 ± 1.81^a^ | 18.49 ± 2.70^a^ |
| 13 | 21.98 ± 1.86^a^ | 18.91 ± 1.14^b^ | 18.46 ± 1.56^b^ | 18.31 ± 2.33^b^ |
| 14 | 21.18 ± 0.97^a^ | 18.76 ± 3.72^a^ | 19.04 ± 2.67^a^ | 18.63 ± 2.12^a^ |
| 15 | 21.28 ± 1.43^a^ | 18.57 ± 2.34^a^ | 18.18 ± 3.98^a^ | 19.01 ± 2.47^a^ |
| 16 | 21.07 ± 1.13^a^ | 18.62 ± 2.21^b^ | 19.27 ± 0.98^ab^ | 18.71 ± 1.30^b^ |
| 17 | 21.39 ± 1.09^a^ | 19.47 ± 1.83^ab^ | 18.84 ± 2.14^b^ | 19.91 ± 1.50^b^ |

^1^ Values are expressed as means ± SD (n = 6, respectively). Bars with different lowercase letters denote significant differences among groups (*p* < 0.05). HFD, fed high fat diet for 17 weeks; HFD+BAA6, fed high fat diet and the final 8 weeks treated with *Bifidobacterium animalis subsp. Lactis* A6; HFD+AKK, fed high fat diet and the final 8 weeks treated with *Akkermansia muciniphila*; ND, fed normal diet for 17 weeks.

Supplementary Table S2. Effects of oral administration of BAA6 or AKK on vitamin intake in HFD-fed mice^1^.

|  | Vitamin intake, g | | | |
| --- | --- | --- | --- | --- |
| Time, weeks | ND | HFD | HFD+AKK | HFD+BAA6 |
| 1 | 0.18 ± 0.02^a^ | 0.19 ± 0.02^a^ | 0.19 ± 0.04^a^ | 0.19 ± 0.04^a^ |
| 2 | 0.19 ± 0.03^a^ | 0.23 ± 0.03^a^ | 0.22 ± 0.02^a^ | 0.22 ± 0.03^a^ |
| 3 | 0.20 ± 0.02^b^ | 0.24 ± 0.02^a^ | 0.24 ± 0.01^a^ | 0.22 ± 0.02^ab^ |
| 4 | 0.19 ± 0.02^b^ | 0.24 ± 0.01^a^ | 0.24 ± 0.01^a^ | 0.24 ± 0.03^a^ |
| 5 | 0.20 ± 0.02^b^ | 0.25 ± 0.04^a^ | 0.25 ± 0.04^a^ | 0.24 ± 0.03^a^ |
| 6 | 0.20 ± 0.01^b^ | 0.25 ± 0.04^a^ | 0.24 ± 0.03^ab^ | 0.24 ± 0.04^ab^ |
| 7 | 0.20 ± 0.02^b^ | 0.24 ± 0.02^a^ | 0.25 ± 0.03^a^ | 0.25 ± 0.02^a^ |
| 8 | 0.20 ± 0.02^b^ | 0.23 ± 0.01^a^ | 0.25 ± 0.03^a^ | 0.24 ± 0.02^a^ |
| 9 | 0.20 ± 0.02^b^ | 0.25 ± 0.04^a^ | 0.24 ± 0.03^a^ | 0.24 ± 0.2^a^ |
| 10 | 0.20 ± 0.01^b^ | 0.24± 0.03^a^ | 0.24 ± 0.03^a^ | 0.24± 0.04^a^ |
| 11 | 0.21 ± 0.01^b^ | 0.24 ± 0.02^a^ | 0.24 ± 0.04^a^ | 0.24 ± 0.03^a^ |
| 12 | 0.21 ± 0.01^a^ | 0.24 ± 0.06^a^ | 0.24 ± 0.02^a^ | 0.24 ± 0.03^a^ |
| 13 | 0.21 ± 0.02^b^ | 0.24 ± 0.01^a^ | 0.24 ± 0.02^a^ | 0.24 ± 0.03^a^ |
| 14 | 0.20 ± 0.01^a^ | 0.24 ± 0.05^a^ | 0.25 ± 0.03^a^ | 0.24 ± 0.03^a^ |
| 15 | 0.20 ± 0.01^a^ | 0.24 ± 0.03^a^ | 0.23 ± 0.05^a^ | 0.25 ± 0.03^a^ |
| 16 | 0.20 ± 0.01^b^ | 0.24 ± 0.03^a^ | 0.25 ± 0.01^a^ | 0.24 ± 0.01^a^ |
| 17 | 0.20 ± 0.01^b^ | 0.25 ± 0.02^a^ | 0.24 ± 0.03^a^ | 0.24 ± 0.2^a^ |

^1^ Values are expressed as means ± SD (n = 6, respectively). Bars with different lowercase letters denote significant differences among groups (*p* *<* 0.05).

Supplementary Table S3. Effects of oral administration of BAA6 or AKK on mineral intake in HFD-fed mice^1^.

|  | Mineral intake, g | | | |
| --- | --- | --- | --- | --- |
| Time, weeks | ND | HFD | HFD+AKK | HFD+BAA6 |
| 1 | 0.18 ± 0.02^a^ | 0.19 ± 0.02^a^ | 0.19 ± 0.04^a^ | 0.19 ± 0.04^a^ |
| 2 | 0.19 ± 0.03^a^ | 0.23 ± 0.03^a^ | 0.22 ± 0.02^a^ | 0.22 ± 0.03^a^ |
| 3 | 0.20 ± 0.02^b^ | 0.24 ± 0.02^a^ | 0.24 ± 0.01^a^ | 0.22 ± 0.02^ab^ |
| 4 | 0.19 ± 0.02^b^ | 0.24 ± 0.01^a^ | 0.24 ± 0.01^a^ | 0.24 ± 0.03^a^ |
| 5 | 0.20 ± 0.02^b^ | 0.25 ± 0.04^a^ | 0.25 ± 0.04^a^ | 0.24 ± 0.03^a^ |
| 6 | 0.20 ± 0.01^b^ | 0.25 ± 0.04^a^ | 0.24 ± 0.03^ab^ | 0.24 ± 0.04^ab^ |
| 7 | 0.20 ± 0.02^b^ | 0.24 ± 0.02^a^ | 0.25 ± 0.03^a^ | 0.25 ± 0.02^a^ |
| 8 | 0.20 ± 0.02^b^ | 0.23 ± 0.01^a^ | 0.25 ± 0.03^a^ | 0.24 ± 0.02^a^ |
| 9 | 0.20 ± 0.02^b^ | 0.25 ± 0.04^a^ | 0.24 ± 0.03^a^ | 0.24 ± 0.2^a^ |
| 10 | 0.20 ± 0.01^b^ | 0.24± 0.03^a^ | 0.24 ± 0.03^a^ | 0.24± 0.04^a^ |
| 11 | 0.21 ± 0.01^b^ | 0.24 ± 0.02^a^ | 0.24 ± 0.04^a^ | 0.24 ± 0.03^a^ |
| 12 | 0.21 ± 0.01^a^ | 0.24 ± 0.06^a^ | 0.24 ± 0.02^a^ | 0.24 ± 0.03^a^ |
| 13 | 0.21 ± 0.02^b^ | 0.24 ± 0.01^a^ | 0.24 ± 0.02^a^ | 0.24 ± 0.03^a^ |
| 14 | 0.20 ± 0.01^a^ | 0.24 ± 0.05^a^ | 0.25 ± 0.03^a^ | 0.24 ± 0.03^a^ |
| 15 | 0.20 ± 0.01^a^ | 0.24 ± 0.03^a^ | 0.23 ± 0.05^a^ | 0.25 ± 0.03^a^ |
| 16 | 0.20 ± 0.01^b^ | 0.24 ± 0.03^a^ | 0.25 ± 0.01^a^ | 0.24 ± 0.01^a^ |
| 17 | 0.20 ± 0.01^b^ | 0.25 ± 0.02^a^ | 0.24 ± 0.03^a^ | 0.24 ± 0.2^a^ |

^1^ Values are expressed as means ± SD (n = 6, respectively). Bars with different lowercase letters denote significant differences among groups (*p* < 0.05).

**Supplementary Table S4. Composition of diets^1^.**

|  | Normal diet | | High fat diet | |
| --- | --- | --- | --- | --- |
| Ingredient | g/kg |  | g/kg |  |
| Casein, 30 Mesh | 189.48 |  | 258.45 |  |
| L-Cystine | 2.84 |  | 3.88 |  |
| Corn Starch | 298.44 |  | 0 |  |
| Maltodextrin 10 | 33.16 |  | 161.53 |  |
| Sucrose | 331.60 |  | 88.91 |  |
| Cellulose, BW200 | 47.37 |  | 64.61 |  |
| Soybean Oil | 23.69 |  | 32.31 |  |
| Lard | 18.95 |  | 316.60 |  |
| Mineral Mix S10026^2^ | 9.47 |  | 12.92 |  |
| DiCalcium Phosphate | 12.32 |  | 16.80 |  |
| Calcium Carbonate | 5.21 |  | 7.11 |  |
| Potassium Citrate, 1 H_2_O | 15.63 |  | 21.32 |  |
| Vitamin Mix V10001^3^ | 9.47 |  | 12.92 |  |
| Choline Bitartrate | 1.89 |  | 2.58 |  |
| FD&C Yellow Dye #5 | 0.05 |  | 0 |  |
| FD&C Blue Dye #1 | 0 |  | 0.06 |  |
|  | g/kg | % energy | g/kg | % energy |
| Protein | 190 | 20 | 262 | 20 |
| Carbohydrate | 673 | 70 | 263 | 20 |
| Fat | 43 | 10 | 349 | 60 |
| Total |  | 100 |  | 100 |
| kcal/g | 3.85 |  | 5.24 |  |

^1^ Formulated and supplied from Research Diets (New Brunswick, US).

^2^ Containing (g/kg mineral mix): sodium chloride, 259; magnesium oxide, 41.9; magnesium sulfate, 7H_2_O, 257.6; ammonium molybdate, 4H_2_O, 0.3; chromium potassium sulfate, 1.925; copper carbonate, 1.05; ferric citrate, 21; manganese carbonate, H_2_O, 12.25; potassium iodate, 0.035; sodium fluoride, 0.2; sodium selenite, 0.035; zinc carbonate, 5.6; sucrose, 399.105.

^3^ Containing (g/kg vitamin mix): retinyl palmitate, 0.8; cholecalciferol, 1.0; all-rac-α-tocopheryl acetate, 10; menadione sodium bisulfite, 0.08; biotin, 2; cyanocobalamin, 1; folic acid, 0.2; nicotinic acid, 0.2; calcium panthotenate, 1.6; pyroxidine-HCl, 0.7; riboflavin, 0.6; thiamin HCl, 0.6; sucrose, 978.42.

Supplementary Table S5. The sequences of primers used in epididymal adipose and liver tissues gene expression.

| Gene | Forward primer (5'→3') | Reverse primer (5'→3') |
| --- | --- | --- |
| *Fas* | ATGCTGTGGATCTGGGCTGTC | CAGTTTCACGAACCCGCCTC |
| *Hsl* | AGACCACATCGCCCACA | CCTTTATTGTCAGCTTCTTCAAGG |
| *Pgc-1α* | AGCCGTGACCACTGACAACGAG | GCTGCATGGTTCTGAGTGCTAAG |
| *Nrf-1* | CGAAAGAGACAGCAGACACG | TTGAAGACAGGGTTGGGTT |
| *Errα* | GGAGTACGTCCTGCTGAAAGCT | CACAGCCTCAGCATCTTCAATG |
| *β-Actin* | GCCAACCGTGAAAAGATGACC | CCCTCGTAGATGGGCACAGT |
| *Ucp-1* | TGGAGGTGTGGCAGTGTTCAT | TGACAGTAAATGGCAGGGGAC |
| *eNos* | TCAGCCATCACAGTGTTCCC | ATAGCCCGCATAGCGTATCAG |
